# Origins of the variability of the electrical characteristics of solution-processed carbon nanotube thin-film transistors and integrated circuits

**DOI:** 10.1039/c8na00184g

**Published:** 2018-10-15

**Authors:** Jun Hirotani, Shigeru Kishimoto, Yutaka Ohno

**Affiliations:** Department of Electronics, Nagoya University Furo-cho, Chikusa-ku Nagoya 464-8603 Japan yohno@nagoya-u.jp; Institute of Materials and Systems for Sustainability, Nagoya University Furo-cho, Chikusa-ku Nagoya 464-8603 Japan

## Abstract

Carbon nanotube (CNT) thin-film transistors based on solution processing have great potential for use in future flexible and wearable device technologies. However, the considerable variability of their electrical characteristics remains a significant obstacle to their practical use. In this work, we investigated the origins of the variability of these electrical characteristics by performing statistical analysis based on spatial autocorrelation and Monte Carlo simulation. The spatial autocorrelation of the on-current decreased with increasing distance on the order of millimetres, showing that macroscopic non-uniformity of the CNT density was one of the causes of the characteristic variability. In addition, even in the local regime where the macroscopic variability is negligible, the variability was greater than that expected based on the Monte Carlo simulation. The CNT aggregation could be attributed to microscopic variability. We also investigated the variability of the properties of integrated circuits such as inverters and ring oscillators fabricated on flexible plastic film. All of the inverters worked well, and their threshold voltage variations were fairly small. As the number of stages in the ring oscillator increased, the yield decreased, although the oscillation frequency variability improved.

## Abstract 2

Carbon nanotube (CNT) thin-film transistors based on solution processing have great potential for use in future flexible and wearable device technologies.

## Graphical abstract

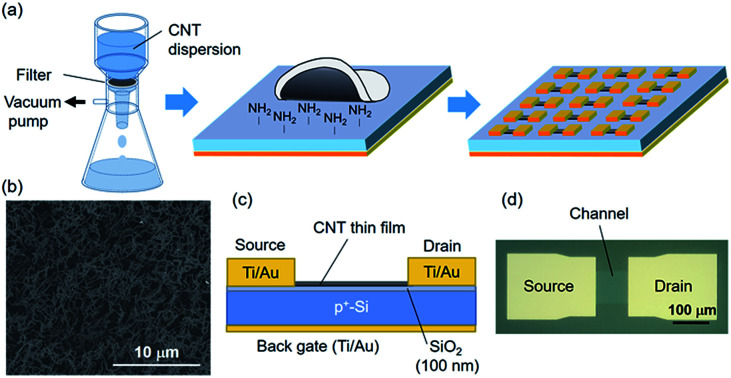

## Introduction

In recent years, flexible electronics have attracted considerable attention due to the wide range of potential applications, from flexible displays^1,2^ to wearable healthcare devices.^3^ Carbon nanotube thin-film transistors (CNT TFTs) are considered to be promising building blocks for flexible electronics because of their remarkable electrical^4^ and mechanical properties.^5^ CNT TFTs are also advantageous in simple fabrication processes such as solution processing.^6–9^

To date, significant efforts have been made to realize high-performance CNT TFTs,^10,11^ medium-scale integrated circuits (ICs)^12^ and random-access memory^13^ based on CNT TFTs, and large-scale complementary circuits using CNT and oxide-semiconductor TFTs.^14^ However, device-to-device variation of the electrical characteristics remains an obstacle to their practical application. For instance, the characteristic variability of CNT TFTs causes operation margin degradation, operation voltage increases, and integration scale limitations in ICs and non-uniformity in the pixel-to-pixel brightness of flat-panel displays.

The variability of the electrical characteristics of CNT TFTs is intrinsically caused by the randomness of the assembled CNT network in the CNT thin-film channel. Two-dimensional percolation theory predicts that, as the number density of CNTs, and hence the number of current paths in the channel, increases, the on-current variability decreases due to the averaging of the currents, which are different for different current paths.^15^ However, as-grown CNTs are mixtures of semiconducting CNTs and metallic CNTs, causing a short-circuit problem in channels with increased CNT number densities, resulting in on/off ratio degradation. The short-circuit probability due to metallic CNTs also increases with decreasing channel length, even though channel length reduction is favourable for obtaining high-performance transistors. There are trade-offs between the uniformity and on/off ratio, as well as between the uniformity and performance in the case of as-grown CNTs.^10,16^

To overcome these trade-offs, the use of high-purity semiconducting CNTs is essential. Recent post-growth purification techniques such as gel chromatography,^17^ density-gradient ultracentrifugation,^18^ DNA-wrapping separation,^19^ and two-phase separation,^20^ have enabled high-purity semiconducting CNTs (s-CNTs) to be obtained. There are several methods of fabricating thin films from s-CNT suspensions, such as drop casting,^21^ immersion coating,^22^ and spray coating.^23^ However, CNTs may easily aggregate during solution-based film formation due to the surface tension of the liquid when the suspension is dried, resulting in additional variation of the device characteristics. Several studies on the characteristic variations of s-CNT-based TFTs have been reported on so far.^8,24,25^ Ohmori *et al.* reported that the characteristic variations can be reduced by using shorter CNTs,^25^ although the carrier mobility may be degraded due to the increase in the number of CNT-to-CNT junctions in the current path. Tian *et al.* achieved wafer-scale fabrication of CNT TFTs with high yield on a 4-inch Si substrate by drop coating.^8^ They also investigated the variation of the device characteristics; however, the cause of the characteristic variations is still not fully understood.

In this work, we studied the origin of the variability of the electrical characteristics of s-CNT TFTs by performing statistical analysis of a large number of devices containing more than 8000 CNT TFTs. Large-area s-CNT thin films were formed *via* suction filtration and transfer. The causes of the characteristic variations were assessed by conducting spatial auto-correlation analysis. We also studied the variability of CNT-based ICs such as inverters and ring oscillators fabricated on flexible plastic film.

## Experimental

**Fig. 1:**
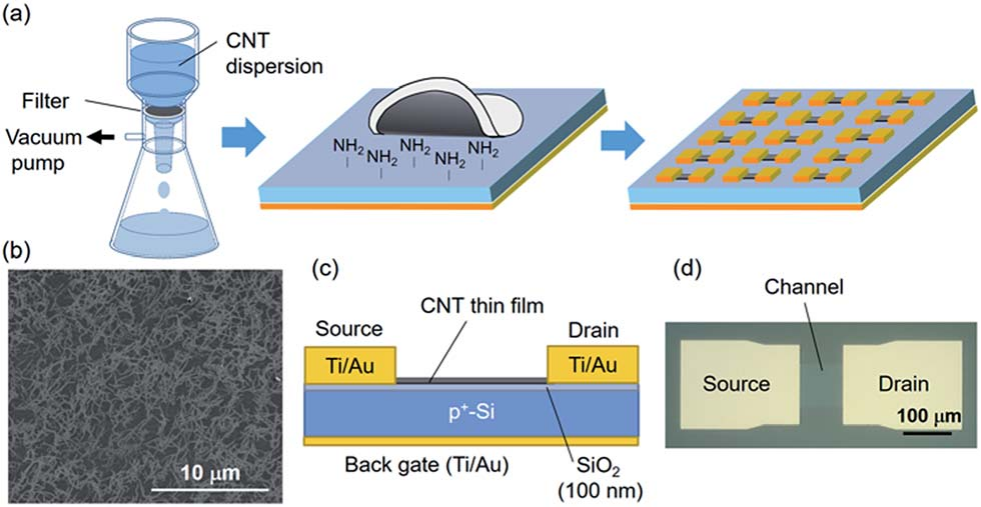
(a) s-CNT TFT fabrication *via* vacuum suction filtration and transfer. An s-CNTs thin film was formed on a membrane filter by suction filtration and was then transferred onto an APTES-functionalized SiO_2_/Si substrate. The device fabrication was completed with source/drain formation and CNT patterning. (b) SEM image of CNT film transferred onto the SiO_2_/Si substrate. (c) and (d) Schematic structure and optical micrograph of a CNT TFT, respectively.

In this study, we employed s-CNTs separated by gel chromatography.^26^ First, we obtained single-walled CNTs synthesized by chemical vapour deposition (KH Chemicals). The purity of the s-CNTs was determined to be 95% based on the absorption spectrum.^27^ The mean diameter (*d*_CNT_) and length (*L*_CNT_) of the s-CNTs were measured to be 1.3 nm based on the optical absorption^28^ and 0.52 μm based on an atomic force microscopy image of individually dispersed s-CNTs on a Si wafer, respectively. The s-CNTs were dispersed in an aqueous solution consisting of a mixture of sodium dodecyl sulfate (0.3 wt%) and sodium cholate (1 wt%). The s-CNT film was formed from the dispersion of 50 mL in volume by vacuum suction filtration with a nitrocellulose-based membrane filter of 47 mm in diameter (VMWP04700, Millipore) at flow rate of ∼0.1 mL s^−1^, as shown in Fig. 1(a). The s-CNT film was transferred onto a heavily doped p-Si substrate with a 100 nm-thick thermally grown SiO_2_ layer and a back gate electrode. The SiO_2_ surface was functionalized by 3-aminopropyltriethoxysilane (APTES, Sigma-Aldrich) prior to the transfer to improve the s-CNT adsorption on the substrate.^29^ In the transfer process, the membrane filter was attached to the substrate and then dissolved with acetone. The sample was immersed in 70 °C water for 1 h to remove the surfactants. We confirmed that the CNTs were successfully transferred onto the target substrate by performing scanning electron microscopy (SEM), as shown in Fig. 1(b).

A schematic of the device structure is shown in Fig. 1(c). The source and drain electrodes were formed *via* photolithography, electron-beam evaporation, and lift-off. Finally, the s-CNTs were patterned by photolithography and oxygen plasma etching. The channel length (*L*) and width (*W*) were held constant at 100 μm. To understand the effect of the CNT number density in the channel on the variability of the electrical characteristics, we prepared CNT films with number densities ranging from 51 CNTs per μm^2^ to 341 CNTs per μm^2^, which corresponded to 2.5*ρ*_th_ − 16*ρ*_th_, where *ρ*_th_ is the two-dimensional percolation threshold given by *ρ*_th_ = 4.24^2^/(π*L*_CNT_^2^).^30^ More than 500 CNT TFTs were produced for each CNT density.

We also investigated the yield and variability of ICs fabricated on a flexible substrate. In this case, bottom-gate CNT TFTs were fabricated on a poly(ethylene naphthalate) (PEN) substrate using the same method as Sun *et al.*,^10^ while a s-CNT thin film was employed as the channel.

## Results and discussion

**Fig. 2:**
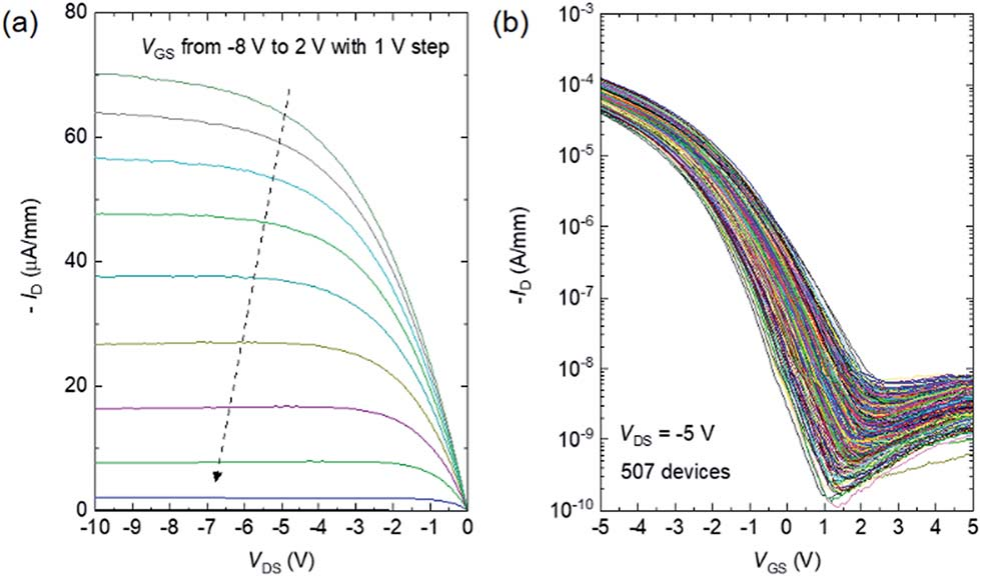
(a) *I*_D_–*V*_DS_ characteristics of a typical CNT TFT with a CNT density of 149 CNTs per μm^2^, (b) *I*_D_–*V*_GS_ characteristics of 507 devices at *V*_DS_ = −5 V.

Fig. 2(a) shows typical drain current (*I*_D_)–drain voltage (*V*_DS_) characteristics of a fabricated CNT TFT with a CNT density of 149 CNTs per μm^2^, exhibiting clear saturation characteristics. Fig. 2(b) depicts the *I*_D_–gate voltage (*V*_GS_) characteristics of 507 devices measured in the saturation region at *V*_DS_ = −5 V. All of the devices exhibit normal p-type transfer characteristics, except for several devices that were defective due to failure during the lithography process. The yield was 99.2% (507/511). The average on/off ratio and mobility were ∼10^5^ and 14.1 cm^2^ V^−1^ s^−1^, respectively. The standard deviation of the on-current (*I*_ON_), which was defined as *I*_D_ in the saturation region at *V*_DS_ = −5 V and *V*_GS_ = −5 V, was fairly small, 21.7%.

**Fig. 3:**
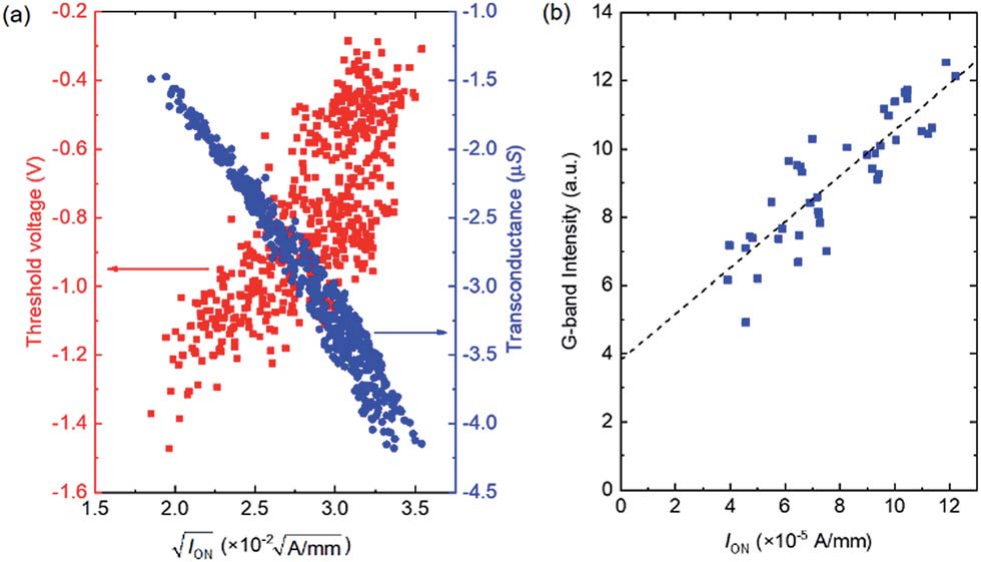
(a) Threshold voltage and transconductance *versus I*_ON_^1/2^ for the 507 devices shown in Fig. 2(b). (b) G-band intensity of Raman scattering *versus I*_ON_ of various CNT TFTs. The broken line was obtained *via* the least squares method.

In general, *I*_ON_ is a function of the threshold voltage (*V*_th_) and transconductance (*g*_m_) according to *I*_ON_ = *g*_m_(*V*_GS_ − *V*_th_)/2, where *g*_m_ = *WμC*(*V*_GS_*− V*_th_)/*L* and *μ* and *C* are the mobility and gate capacitance, respectively. The variation of *I*_ON_ was assessed by evaluating the correlations between *I*_ON_ and *V*_th_ and between *I*_ON_ and *g*_m_. Fig. 3(a) shows *V*_th_ and *g*_m_ as functions of the square root of *I*_ON_ for the 507 devices shown in Fig. 2(b). Here, *V*_th_ was measured by extrapolating a linear fit of the *I*_D_^1/2^–*V*_GS_ characteristics. The correlation coefficients between *I*_ON_^1/2^ and *V*_th_ and between *I*_ON_^1/2^ and *g*_m_ are 0.760 and −0.977, respectively, showing that both *V*_th_ and *g*_m_ variations caused *I*_ON_ variations. However, there is a stronger correlation between *I*_ON_ and *g*_m_. The plausible cause of the variation of *g*_m_ is CNT density non-uniformity, *i.e.* both *C* and *μ* are affected by the CNT density.

The correlation between *I*_ON_ and the CNT density was also investigated by performing Raman scattering spectroscopy. In the Raman measurements, the diameter of the excitation laser on the sample was set to 140 μm, and the Raman signal was taken from whole region of the TFT channel, so the Raman intensity was proportional to the amount of CNTs in the channel. Fig. 3(b) shows *I*_ON_*versus* the G-band intensity of the Raman scattering for 42 devices. A clear correlation is evident between *I*_ON_ and the G-band intensity with a correlation efficient of 0.87, showing that the *I*_ON_ variation was primarily caused by the variation of the amount of CNTs in the channel.

**Fig. 4:**
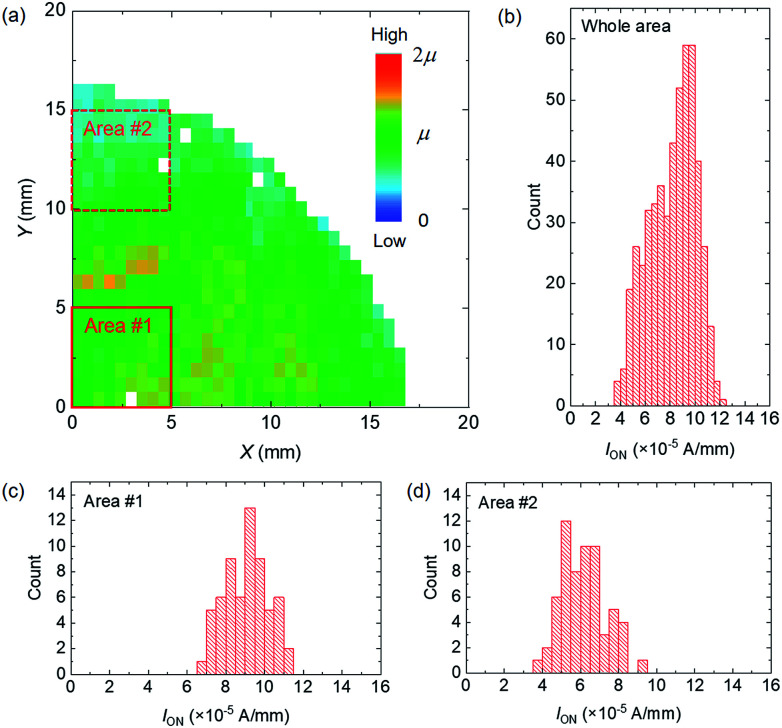
(a) Spatial distribution of on-current for the 507 devices shown in Fig. 2(b). The average on-current is denoted as *μ* in the colour scale. *V*_DS_ = *V*_GS_ = −5 V. On-current histograms for (b) the whole device and (c) area #1 and (d) area #2 surrounded by red squares in (a).

Fig. 4(a) and (b) present the spatial distribution and histogram of *I*_ON_, respectively, for 507 devices contained in a quarter of the sample. A macroscopic distribution with dimensions of several millimetres can be seen in the *I*_ON_ map. In the small 5 × 5 mm^2^ areas labelled area #1 and area #2, which are surrounded by red squares in Fig. 4(a), the standard deviations of the *I*_ON_ distribution were found to be 12.2% (Fig. 4(c)) and 18.9% (Fig. 4(d)), which are less than overall variation mentioned before.

**Fig. 5:**
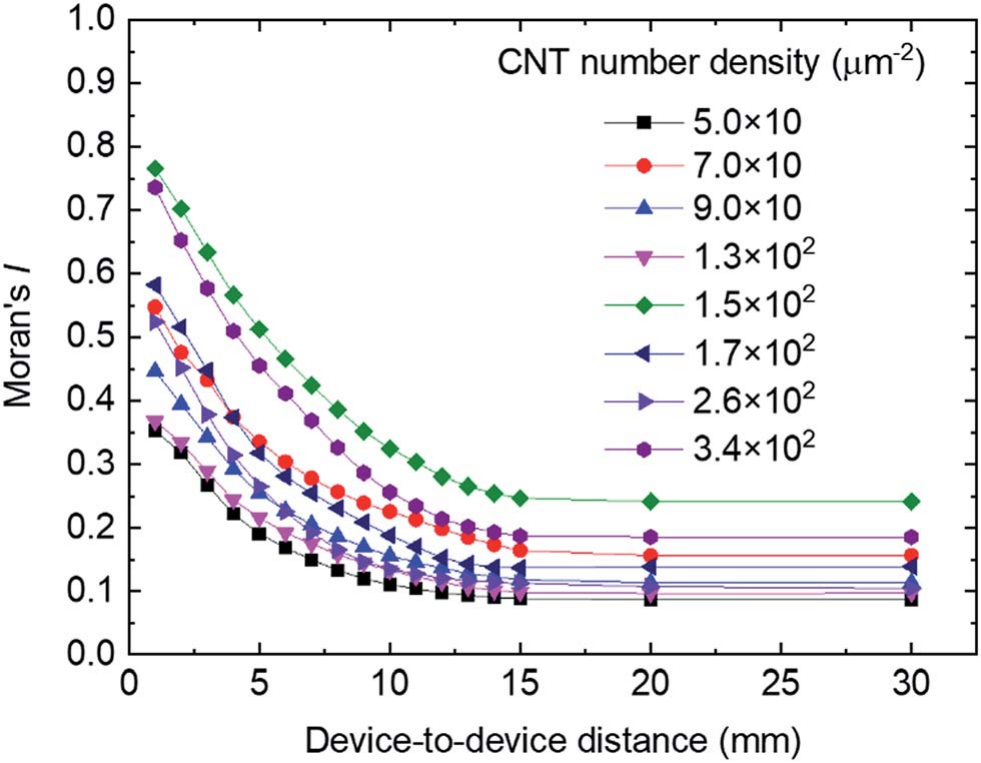
Spatial autocorrelation (Moran's *I*) as a function of distance between CNT TFTs.

To determine the potential uniformity without macroscopic variation, we adopted spatial autocorrelation analysis. The spatial autocorrelation (Moran's *I*), which shows the similarities between distant devices, is given by^31^1
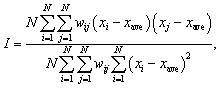
where *N* is the number of samples, *x*_*i*_ is *I*_ON_ for a device with an index *i*, *x*_ave_ is the average *I*_ON_, and *i* and *j* are indices. We employed an inverse-distance weight factor, *w*_*ij*_ = 1/*d*_*ij*_, where *d*_*ij*_ is the distance between two devices with indices of *i* and *j*. The spatial autocorrelation is shown as a function of the device-to-device distance in Fig. 5 for various CNT number densities. With increasing distance between the devices, Moran's *I* decreases, showing that neighbouring TFTs have more similar *I*_ON_ values than distant ones do, and the local variation of *I*_ON_ is less than the macroscopic variation. The distance that decreased Moran's *I* by half was about 5 mm. Then, we evaluated the local variations in the 5 × 5 mm^2^ areas to exclude the effects of the macroscopic variations, and the smallest variation of *I*_ON_ was determined to be 4.2% for a CNT density of 149 μm^−2^.

**Fig. 6:**
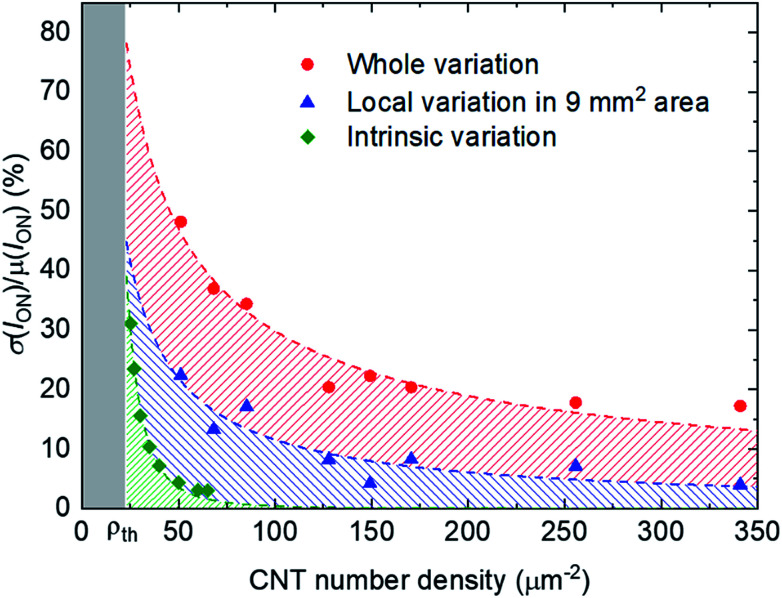
Standard deviation of *I*_ON_ divided by the average *I*_ON_ value as a function of the number density of CNTs in the channel. The red circles and blue triangles are the experimental data obtained for whole devices and devices in a local area of 9 mm^2^, respectively. The green diamonds represent the data obtained by Monte Carlo simulation. The hatched regions show the macroscopic (red), microscopic (blue), and intrinsic (green) variations.

The standard deviation of *I*_ON_, *σ*(*I*_ON_), divided by the average *I*_ON_, *μ*(*I*_ON_), is shown as a function of the CNT number density in Fig. 6. The red dots and blue triangles represent the experimentally obtained variations for the overall sample and a 9 mm^2^ area, respectively. The green squares depict the variations obtained by performing a Monte Carlo simulation. In the simulation, conductive sticks were randomly dispersed in the channel area and *I*_ON_ was calculated by assuming that the contact resistances of the CNT-to-CNT junctions dominated the channel resistance rather than the resistances of the CNTs. We also assumed *W* = *L* = 100 μm, *L*_CNT_ = 0.5 μm, an s-CNT purity of 95%, and a CNT-to-CNT junction resistance of 100 kΩ in the on state. The simulated variations intrinsically originate from the randomness of network-like CNT thin film. This intrinsic variations decrease with increasing CNT density. In the experimental results, however, we obtained two types of variations in addition to the intrinsic variations: the microscopic variations observable in the 9 mm^2^ area indicated by the blue hatched area in the Fig. 6, which were probably caused by the aggregation of CNTs, as can be seen in the SEM results in Fig. 1(b); and macroscopic variations, as indicated by the red hatched area, which correspond to the *I*_ON_ variations observable in Fig. 4(a).

These additional variations were not reduced by increasing the CNT density. With a CNT density of 149 μm^−2^, ∼18% (∼6%) of the *I*_ON_ variations were attributed to the macroscopic (microscopic) variations; thus, the macroscopic variations mainly caused the *I*_ON_ variations in this work. The macroscopic variation of *I*_ON_ probably resulted from the film formation *via* suction filtration, in which the CNT suspension is likely to flow through the membrane filter unevenly due to the surface tension of the droplets on the drain side of the filter. The droplets of filtrated dispersion drop off from some particular sites of the membrane filter, which may cause the biased flow of the dispersion through the membrane filter *via* the surface tension of the droplets. Controlling the drop-off sites on the membrane filter would be a key to improve the uniformity in the macroscopic scale. In order to reduce the microscopic CNT aggregations, the control of CNT bundling is important. In fact, it was observed from atomic force microscopy that the CNTs were bundled to be 3–4 nm in bundle size. An optimization of CNT dispersion conditions is necessary.

**Fig. 7:**
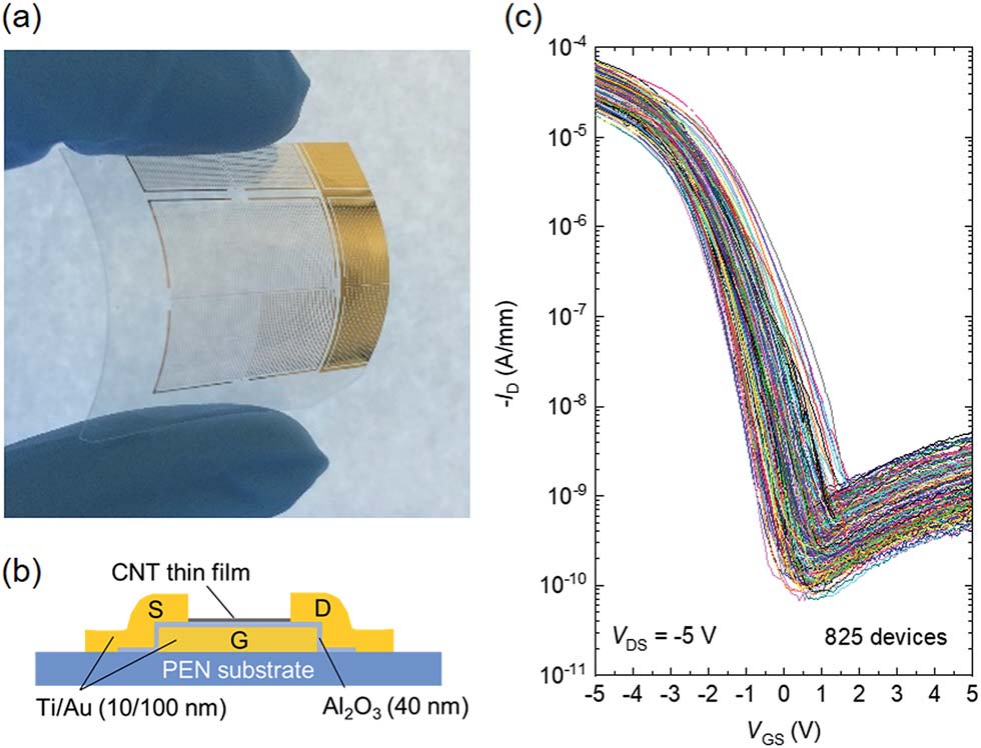
Flexible CNT TFTs. (a) Photograph, (b) schematic device structure, and (c) transfer characteristics of 825 CNT TFTs at *V*_DS_ = −5 V.

Finally, we investigated the impact of the variability of the TFT characteristics on the yield and variation of logic ICs such as inverters and ring oscillators. Fig. 7(a) and (b) show a photograph and schematic of the device structure of a CNT TFT fabricated on a plastic film. Bottom-gate CNT TFTs were fabricated on a PEN substrate. The gate insulator was 40 nm-thick Al_2_O_3_ deposited by atomic layer deposition. *W*/*L* was 100/100 μm. Fig. 7(c) shows the transfer characteristics of 825 devices. The TFT yield was found to be 95.0% (825/868), and the *I*_ON_ variation was 27.2%, which is comparable to that on the Si substrate.

**Fig. 8:**
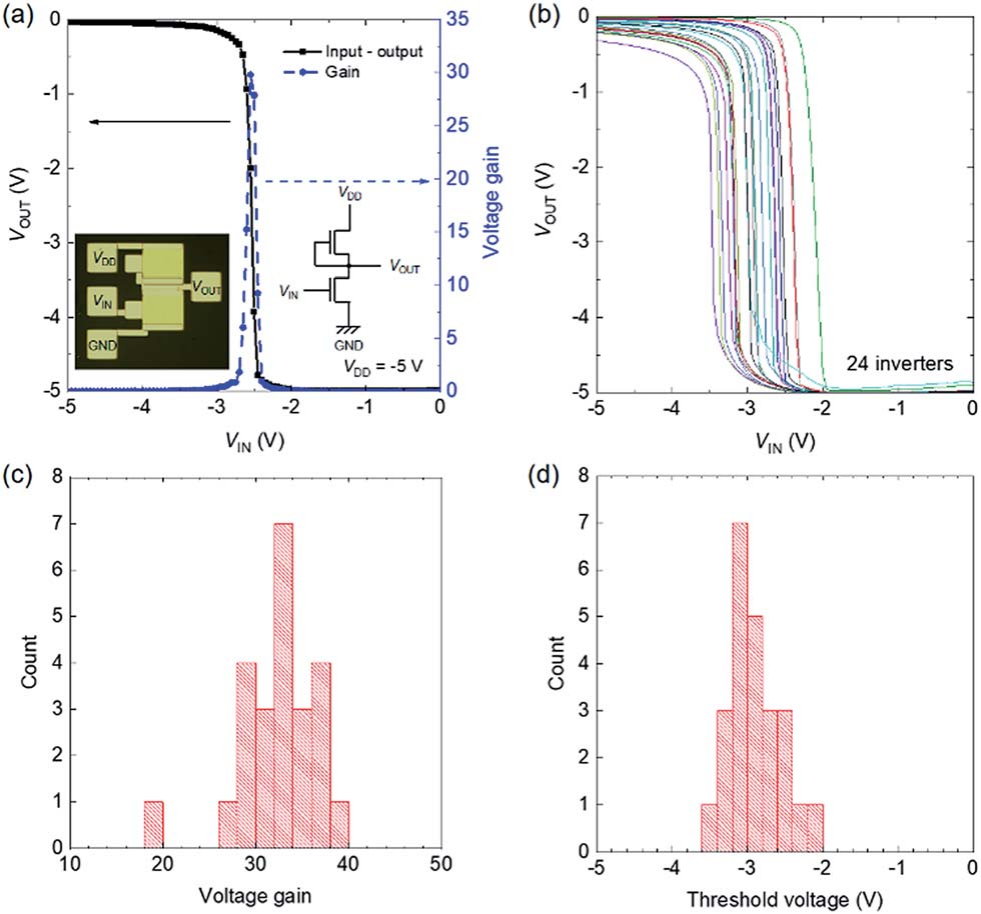
Flexible CNT inverters. (a) Transfer characteristics and voltage gain of an inverter. Insets: photograph and circuit diagram. (b) Transfer characteristics and histograms of the (c) voltage gain and (d) threshold voltage of 24 inverters.

We fabricated inverters with an enhancement/depletion configuration, as shown in the inset of Fig. 8(a). The load TFT was slightly doped with tetrafluoro-tetracyano quinodimethane to shift the threshold voltage into depletion mode. Fig. 8(a) shows the input–output characteristics of an inverter operated at *V*_DD_ = −5 V. Clear inverter operation was obtained with input–output voltage matching. The threshold voltage is −2.6 V, which is close to the ideal value of *V*_DD_/2. The voltage gain is as high as 30.

Fig. 8(b) shows input–output characteristics of 24 inverters. All of the inverters worked successfully, with an average high voltage gain of 32 and logic threshold voltage of 2.9 V, as shown in the histograms in Fig. 8(c) and (d), respectively. The logic threshold voltage distribution is fairly small compared to those reported recently.^32^ The logic threshold voltage of an inverter is determined by the difference between the *V*_T_ values of two transistors in an ideal case; hence, the *V*_T_ distributions of transistors directly influence the logic threshold voltage variability of inverters. In the present case, however, the logic threshold voltage variability (∼1.5 V difference between the maximum and minimum values) was larger than the distribution of the *V*_T_ values of the TFTs (∼0.9 V, not shown). In the present case, the *g*_m_ values of the TFTs were widely distributed due to the non-uniformity of the CNT density, as described before, so the logic threshold voltage was also scattered.

**Fig. 9:**
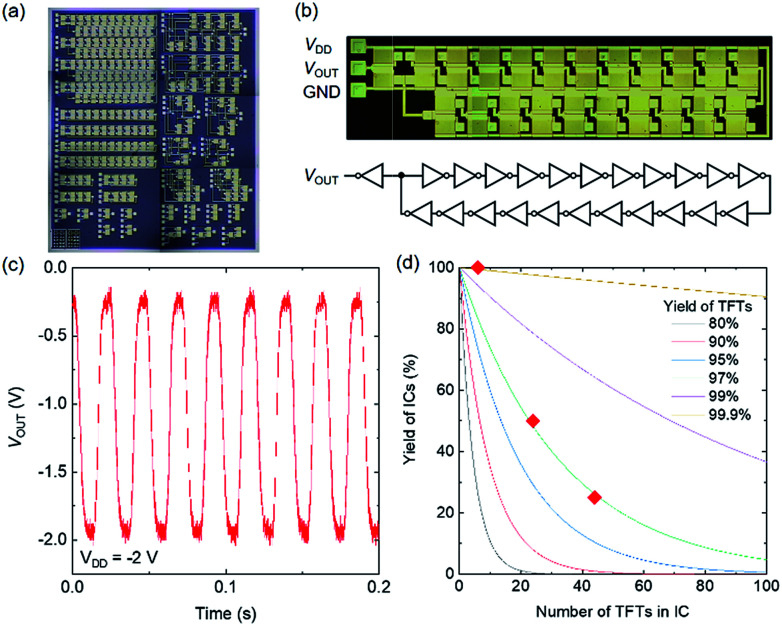
Flexible CNT ring oscillators. (a) Photograph of fabricated ICs. (b) Photograph and circuit diagram of a 21-stage ring oscillator with an output buffer. (c) Output waveform of the 21-stage ring oscillator. (d) Ring oscillator yield as a function of the number of TFTs in the circuit.

In addition, ring oscillators (with 3, 11, and 21 stages) were fabricated on a PEN substrate, as shown in Fig. 9(a). A photograph and circuit diagram of the 21-stage ring oscillator are provided in Fig. 9(b). The output of the buffer amplifier was measured with an oscilloscope *via* an instrument amplifier with high input impedance. A typical oscillation waveform is shown in Fig. 9(c). The ring oscillators exhibit oscillations at *V*_DD_ as low as 2 V due to the local uniformity of the TFT characteristics. The oscillation frequency is 42.5 Hz, corresponding to a switching time of 56 ms for an inverter. The yield of each type of fabricated ring oscillator is shown in Fig. 9(d) as a function of the number of TFTs in the ring oscillator. All of the 3-stage ring oscillators worked; however, the yield decreases as the number of TFTs in the ring oscillator increases, reaching 50% for the 11-stage oscillator (24 TFTs) and 25% for the 21-stage oscillator (44 TFTs). The solid curves in Fig. 9(d) show the calculated IC yields for various TFT yields, *y* = *x*^*N*^, where *x*, *y*, and *N* represent the TFT yield, IC yield, and number of TFTs in the IC, respectively. The yield curve of the fabricated ring oscillator can be fitted by the calculated yield curve when the TFT yield is 97%. Therefore, the ring-oscillator yield degradation is dominated by the TFT yield rather than by the variability of the TFT characteristics.

The variability of the TFT characteristics, however, directly affected the operation speed of the ring oscillators. The delay time (*τ*) per stage ranged from 0.81 ms per stage to 2.8 ms per stage, from 0.98 ms per stage to 3.3 ms per stage, and from 0.53 ms per stage to 0.57 ms per stage for the 3-, 11-, and 21-stage oscillators, respectively. The variation of *τ* decreased as the number of stages in the ring oscillator increased. The standard deviation of *τ* divided by the average value was 44%, 39%, and 4.3% for the 3-, 11-, and 21-stage oscillators, respectively. The *τ* value of an inverter is approximately given by *τ* = *WL*(*C*_GS_ + *C*_p_)/*g*_m_, where *C*_GS_ is the channel capacitance and *C*_p_ is parasitic capacitance attributed to the overlaps between the gate and source/drain electrodes. Therefore, the *g*_m_ variation directly affects the *τ* variation. Note that *g*_m_ is proportional to *C*_GS_ and the variations of *g*_m_ and *C*_GS_ may cancel one another, causing *τ* not to be affected; however, this is not the case for the present devices because *C*_p_ (∼6 pF) was twice as large than *C*_GS_ (∼3 pF) in the present device. As the number of stages in a ring oscillator increases, the oscillation frequency variability can decrease because of the averaging effect of a series connection of inverters.

## Conclusion

The origins of the variability of the electrical characteristics of CNT TFTs were investigated statistically in this study. The *I*_ON_ distribution exhibited a strong correlation with *g*_m_ rather than *V*_T_, showing that the non-uniformity of the CNT density primarily caused the variability of the TFT characteristics. Spatial autocorrelation analysis revealed that there was millimetre-scale, macroscopic non-uniformity in the CNT density. We also found that even in the local regime where the macroscopic variability was negligible, the variability was larger than that expected from the Monte Carlo simulation. The CNT aggregation caused during CNT film fabrication could be attributed to the microscopic variability. It was expected that by eliminating the macroscopic variations, the *I*_ON_ variations could be reduced to 4.2% within a 9 mm^2^ area. We also investigated the variability of the properties of ICs such as inverters and ring oscillators fabricated on flexible plastic film. All 24 inverters worked well, and their logic threshold voltage variations were fairly small. As the number of stages in the ring oscillator increased, the ring oscillator yield decreased; however, the oscillation frequency variability was improved due to the averaging effect of the series connection of inverters. Although the carrier mobility of 14.1 cm V^−1^ s^−1^ would be satisfactory for some applications such as a backplane of flexible e-papers and low frequency-band radio frequency identification (RFID) tags, an improvement of uniformity of CNT thin films is a key issue to be addressed for the practical applications. Our statistical analysis and results offer an effective way to investigate origins of variability of CNT TFTs and ICs.

## Conflicts of interest

There are no conflicts to declare.

## Supplementary Material
